# Recent Progress in Fourier Transform Infrared (FTIR) Spectroscopy Study of Compositional, Structural and Physical Attributes of Developmental Cotton Fibers 

**DOI:** 10.3390/ma6010299

**Published:** 2013-01-22

**Authors:** Yongliang Liu

**Affiliations:** Cotton Structure & Quality Research Unit, SRRC, ARS, USDA, 1100 Robert E. Lee Blvd., New Orleans, LA 70124, USA; E-Mail: yongliang.liu@ars.usda.gov; Tel.: +1-504-286-4455; Fax: +1-504-286-4217

**Keywords:** cotton fiber, cellulose, crystalline and amorphous cellulose, fiber maturity, fiber crystallinity, FTIR, attenuated total reflection, ATR

## Abstract

Cotton fibers are natural plant products, and their end-use qualities depend on their stages of development. In general, the quantity of cellulose in cotton fibers increases rapidly, thus it leads to compositional, structural and physical attribute variations among the fibers with shorter and longer growth periods. This article discusses recent progress in applying the Fourier transform infrared (FTIR) spectroscopic technique to characterize these differences, to discriminate immature fibers from mature fibers, to assess fiber maturity and crystallinity and also to unravel the band assignments in crystalline and amorphous celluloses. The results were achieved through the use of various strategies, including wet chemical analysis, principal component analysis (PCA), simple algorithm development, two-dimensional correlation analysis and other independent fiber tests. Of particular interest is that, in general, immature fibers might have the characteristics of less than 21–28 dpa, *M*_IR_ < 0.58 (in the maturity range of 0 to 1.0) and *CI*_IR_ < 42% (in the crystallinity range of 0 to 100%).

## 1. Introduction

Cellulose I (β 1→4 linked glucose residues) is a major compositional component in mature cotton fibers. Its quantity impacts the end-use qualities for yarn and fabric products. In general, cotton fiber cellulose development is considered to include at least four overlapping, but distinctive, phases: initiation, primary wall formation (elongation), secondary cell wall thickening (cellulose synthesis) and maturation [1]. The day of flowering is referred to as anthesis, and the term “days post anthesis” (dpa) is commonly used to describe the cotton fiber growth. The fiber cells initiate at 0 dpa and then elongate to reach a fiber length of 22~35 mm within 20–25 dpa. The secondary cell wall synthesis starts around 15 to 22 dpa and continues for an additional 30 to 40 days until maturation, when the fibers dehydrate and collapse into flattened and twisted ribbons. Such a fiber evolution suggests a number of essential changes in fiber chemical compositions, structures and physical properties with various stages of development. The word “maturity” has been utilized to reflect the degree of fiber development or thickening of the secondary cell wall of fibers.

**Figure 1:**
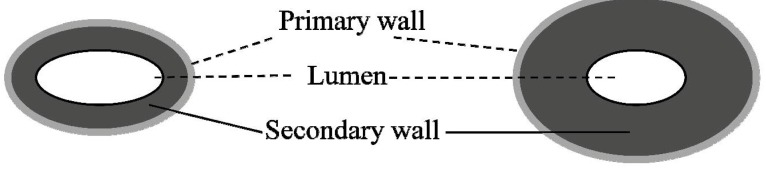
Schematic of immature (left) and mature (right) single fiber cross-sections.

Regarding the degree of fiber maturity, cotton fibers are simply classified into two categories of immature and mature fibers [2]. A typical cross-section overview of an immature (left) and a mature (right) single fiber is shown in Figure 1. Obviously, the ratio of the secondary wall to the total area of the primary wall and lumen increases with the secondary wall thickening (or fiber maturity). Commonly, mature fibers are composed of mostly cellulose (88.0%–96.5%), followed by such noncellulosic constituents as proteins (1.0%–1.9%), waxes (0.4%–1.2%), pectins (0.4%–1.2%), inorganics (0.7%–1.6%) and other substances (0.5%–8%) [1]. In contrast to mature fibers, immature (or low maturity) fibers contain less cellulose and more noncellulosic components. The presence of a large amount of immature fibers in commercial cottons has been found to cause entanglement during mechanical processing and also to affect the desired color appearance in dyed yarn and finished fabric products [3,4]. 

Compositional and structural differences between immature and mature fibers, as well as their physical properties and end-use qualities, have been investigated considerably over the years by diversified and comprehensive techniques, including wet chemistry, microscopy, X-ray diffractometry, molecular spectroscopy and well-defined fiber testing methods in the cotton industry [1,2,5,6,7,8,9,10,11,12,13,14]. Given the obvious distinctions in compositions and structures between mature and immature fibers, appropriate optical and physical means are very successful in reflecting the differences of targeted properties between two types of fibers. Among the techniques, attenuated total reflection (ATR) sampling device based Fourier transform infrared (ATR-FTIR) spectroscopy could be a potential consideration, as it requires minimal sample preparation, permits routine analysis at both laboratory and on-field environments and is easy to operate. It is expected that this technique could be evolved as an effective diagnostic in monitoring cellulose biosynthesis and assessing cotton fiber quality, processing and end-use performance.

## 2. Results and Discussion

### 2.1. Cellulose Formation and Phase Transition

It is well-known that glucose units form cellulose macromolecules through polymerization reaction, during the period of cotton fiber development. This biogenesis process results in a co-existence of fiber cellulose and numerous non-cellulosic components in cotton fibers [1,15].

Recently, Abidi *et al.* [11] determined the amounts of cellulose and four major sugars (including sucrose, glucose, fructose and galacturonic acid) in developmental fibers and compared the changes in their contents between two cotton cultivars (*Gossypium hirsutum L.* cv. TX19 and TX55). As anticipated, percentages of four sugars decrease significantly for two cultivars during the fiber development, while the amount of cellulose increases as a result of secondary cell wall development. Noticeably, TX19 fibers were observed to have a rapid cellulose synthesis from 10.7% to 80.4% between 14 and 24 dpa, with a clear increase at 18 dpa (56.9%). Relatively, cellulose content in developmental TX55 fibers is 9.1% between 10 and 20 dpa, raises to 34.1% at 21 dpa and to 68.3% at 24 dpa, before staying nearly unchanged between 27 and 56 dpa. Earlier start of cellulose synthesis in TX19 fibers (18 dpa) than in the TX55 cultivar (24 dpa) could have a significant impact on fiber maturity at the end of the growing season. They attributed the six day difference to more elevated enzymatic activities in TX19 fibers than in TX55 fibers. 

As a complementary and independent approach, Abidi *et al.* [12] applied the ATR-FTIR spectroscopic tool to investigate the structural changes during cotton cellulose formation. By analyzing the IR bands at 3335, 3280, 2918, 2850, 1733, 1627, 1534, 1236, 900 and 710 cm^−1^, they monitored the dpa-dependent IR intensity variations between two cultivars (TX19 *vs.* TX55). One of the notable views was to examine the relationships between the 1627 cm^−1^ band (adsorbed water) or the 710 cm^−1^ band (CH_2_ rocking vibration in cellulose I*_β_*) against fiber growth. For fibers from the TX19 cultivar, the amount of adsorbed water decreases between 10 and 18 dpa, and no significant changes are observed after 19 dpa; while intensity of the 710 cm^−1^ band increases linearly between 10 and 30 dpa, and no major change is noticed between 30 and 56 dpa. Among TX55 fibers, the amount of adsorbed water decreases linearly until the fibers reach 24 dpa, and a major intensity change of the 710 cm^−1^ band happens only at 21 dpa. Probably, the reduction of the adsorbed water amount could originate from the decrease of the surface area and the minimized accessibility of water molecules to the internal hydroxyl groups, mostly due to the formation of cellulose macromolecules that induce the cellulose re-organization and also increase the ordered (or crystalline) cellulose portion from shorter to longer dpa fibers.

Principal component analysis (PCA) is a very effective variable reduction technique for spectroscopic data. It decomposes the spectra into mathematical spectra (called loading vectors, factors, principal components, *etc.*) that represent the most common variations to all data. It has been used as a tool to interpret the samples on the basis of both their spectral characteristics and their logical assignment. Abidi *et al.* performed PCA processing of respective ATR-FTIR spectra from TX19 and TX55 cultivars [12]. For fibers from the TX19 cultivar, two groups of spectra (or samples) were identified: group 1 includes the spectra of fibers at 10, 14 and 17 dpa with negative PC1 (the first principal component) scores, and group 2 includes the spectra of fibers from 18 to 56 dpa with positive PC1 scores. Among TX55 fibers, two clusters were recognized, with cluster 1 representing the spectra of fibers from 10 to 21 dpa and having negative PC1 scores, whereas cluster 2 includes the spectra of fibers from 24 to 56 dpa and possessing positive PC1 scores. Therefore, they concluded different transition phases between two cultivars, that is, the transition occurs from 17 to 18 dpa for TX19 fibers and between 21 and 24 dpa for TX55 fibers. The finding was well verified by separated measurements of sugar and cellulose contents from established analytical protocols [11].

### 2.2. Discrimination of Immature and Mature Fibers and Assessment of Cellulose Maturity

**Figure 2:**
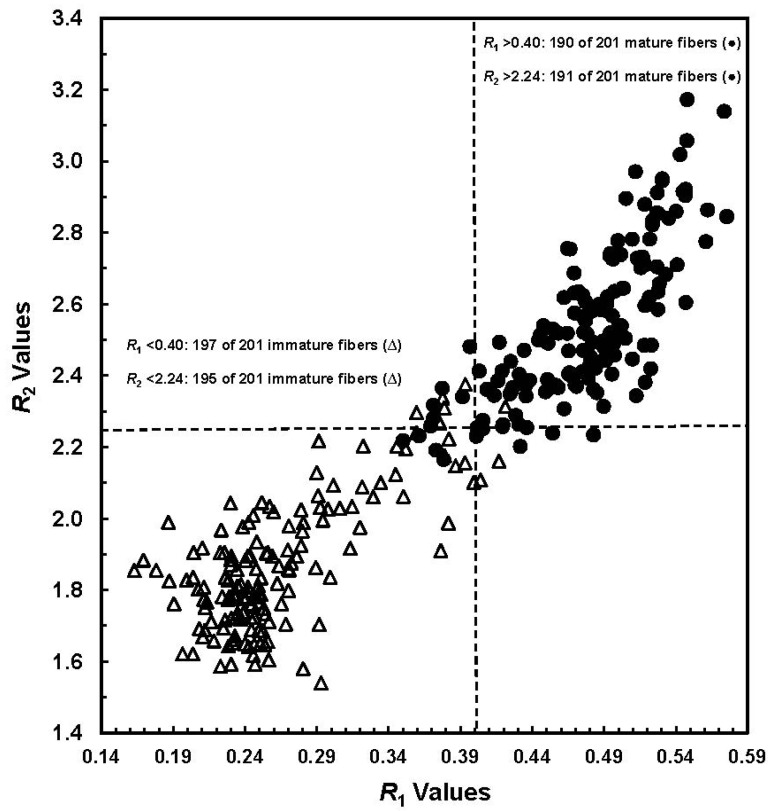
Plot of *R*_1_ values *vs.* *R*_2_ values from a total of 402 manually selected seed cottons (Reprinted with permission from [16]. Copyright 2011 SAGE).

Mature and immature fibers are determined easily from conventional microscopy, but the degree of maturity is difficult to assess. This is because the microscopic procedure is subjective and depends on one’s judgment to assign the fibers into the appropriate class of either immature or mature fibers. On the ATR-FTIR spectral intensity differences between immature and mature fibers, Liu *et al.* determined the key wavelengths first and then developed two simple algorithms (*R*_1_ and *R*_2_) for their discrimination [16]. They observed that the *R*_1_ values increase with *R*_2_ values among a data set consisting of 402 seed bolls (Figure 2). With an *R*_1_ threshold value at 0.40, 197 of 201 (98.0%) immature fibers and 190 of 201 (94.5%) mature samples were correctly classified. By setting an *R*_2_ threshold value at 2.24, only six immature fibers and 10 mature samples were misidentified, yielding an overall 96% accuracy in their correct differentiation. 

**Figure 3:**
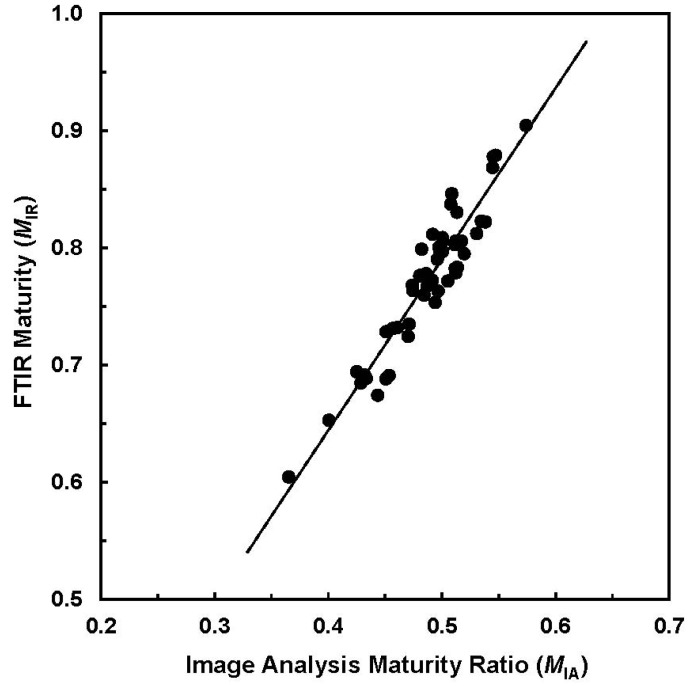
Relationship of *M*_IA_ from image analysis (IA) procedure *vs.* *M*_IR_ from attenuated total reflection (ATR) sampling device based Fourier transform infrared (ATR-FTIR) measurement (R^2^ = 0.894) (Reprinted with permission from [16]. Copyright 2011 SAGE).

Further, they proposed a formula to estimate the degree of cotton cellulose maturity (*M*_IR_) by representing the *R*_1_ values. In this concept, the *M*_IR_ values of 0.0 and 1.0 were assigned to the most immature and mature fibers, respectively. Thus, immature fibers whose *R*_1_ < 0.40 correspond to a *M*_IR_ < 0.58 in the maturity range of 0 to 1.0, and *vice versa*. In order to validate the efficiency of accessing the *M*_IR_ from direct ATR-FTIR measurement, cotton fibers with various maturity readings as determined from traditional image analysis (IA) and advanced fiber information system (AFIS) were taken. Within small and selected samples, strong correlations between *M*_IR_ against referenced IA and AFIS maturity readings were reported. Figure 3 provides a relationship between two maturity measurements (IA against ATR-FTIR) for the selected 50 samples.

### 2.3. Determination of Cellulose Crystallinity

Numerous hydroxyl groups in fiber cellulose are involved in complicated intra- and inter-molecular hydrogen bonds, which yield high-order (crystalline) and low-order (amorphous) regions. Crystallinity index (*CI*) has been used to describe the relative amount of crystalline portion in a simple two-phase model (crystalline *vs.* amorphous). In practice, *CI* of cotton fiber cellulose is determined predominantly by a curve-fitting process that extracts individual crystalline peaks from the X-ray diffraction (XRD) intensity profile. In general, XRD determination of cellulose *CI* provides a qualitative or semi-quantitative evaluation of the amounts of either crystalline or amorphous components in a sample [17]. Hence, appropriate cellulose standards are desired to calibrate or validate the XRD measurement. However, these absolute standards are not easy to be prepared or obtained.

In the preceding ATR-FTIR study of immature and mature cotton fibers [16], the algorithm *R*_2_ was linked with the relative amount of *I*_β_ to *I*_α_ crystal form. Similar to the conception of representing the *R*_1_ value, the *R*_2_ values were converted to *CI*_IR_ [18]. If the most immature and mature fibers were assigned the *CI*_IR_ value of 0% and 100%, respectively, the corresponding *R*_2_ values should be the smallest and largest ones in Figure 2. In turn, immature fibers with *R*_2_ < 2.24 might have *CI*_IR_ < 42%, and mature fibers with *R*_2_ > 2.24 have *CI*_IR_ of >42%. 

**Figure 4:**
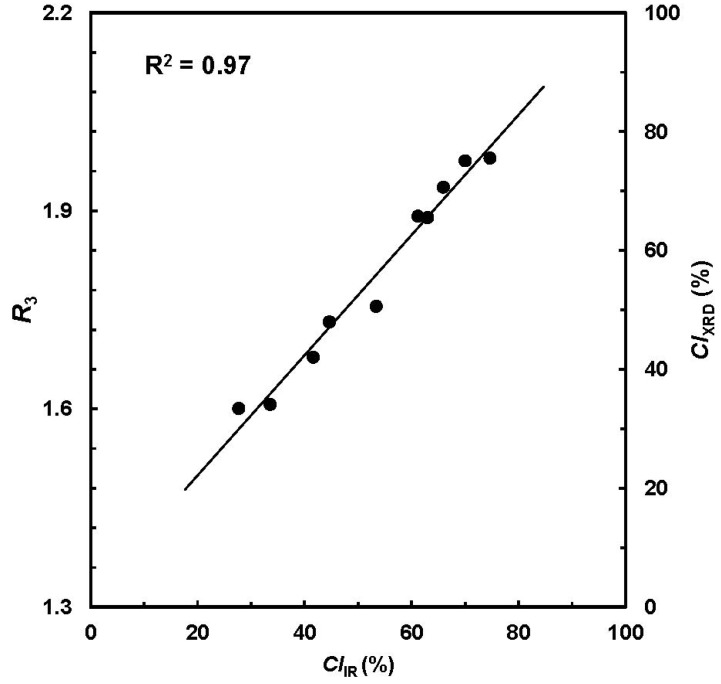
Plot of *CI*_IR_ from ATR-FTIR procedure *vs.* *CI*_XRD_ from X-ray diffraction (XRD) measurement. (Reprinted with permission from [18]. Copyright 2012 Ingentaconnect)

Along with the idea identical to simple algorithms for ATR-FTIR spectral analysis, Liu *et al.* developed a four-band ratio (*R*_3_) that related the XRD intensity with the crystalline information [18]. Relationship between the *CI*_IR_ from ATR-FTIR spectra and *R*_3_ from XRD pattern showed a high correlation of R^2^ > 0.90 (Figure 4). Next, the *R*_3_ values were converted into respective *CI*_XRD_ readings in the range of 0% to 100%, which is depicted as a secondary vertical axis in Figure 4.

**Figure 5:**
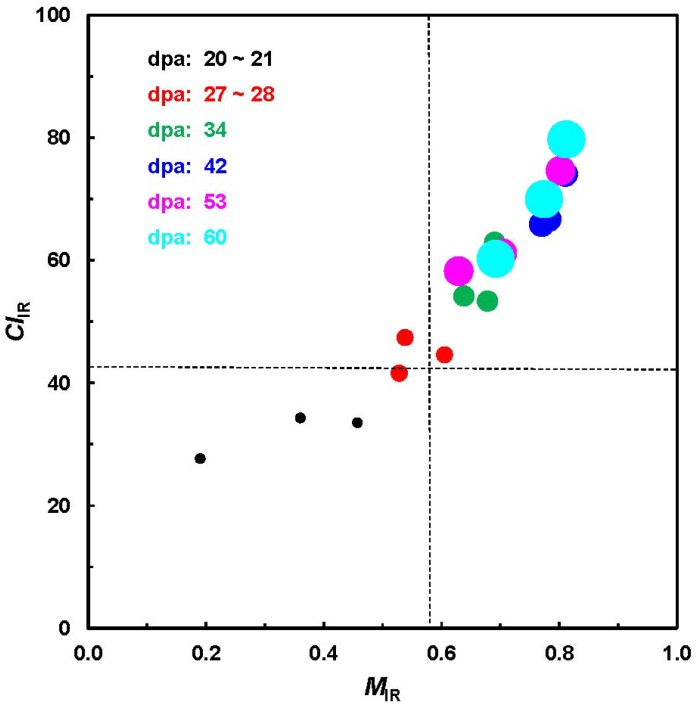
Relationship of *M*_IR_ against *CI*_IR_ values from ATR-FTIR procedure.

As one implementation, Figure 5 shows the relationship between *M*_IR_ and *CI*_IR_ from a total of 18 cotton bolls, representing three varieties and six growth periods from the unique ATR-FTIR measurement. In the line of expectation, both *M*_IR_ and *CI*_IR_ increase from shorter to longer dpa for all varieties. The pattern in Figure 5 is very similar to that in a previous investigation (Figure 2) and also reveals a relatively clear separation among the fibers with differing dpa.

**Figure 6:**
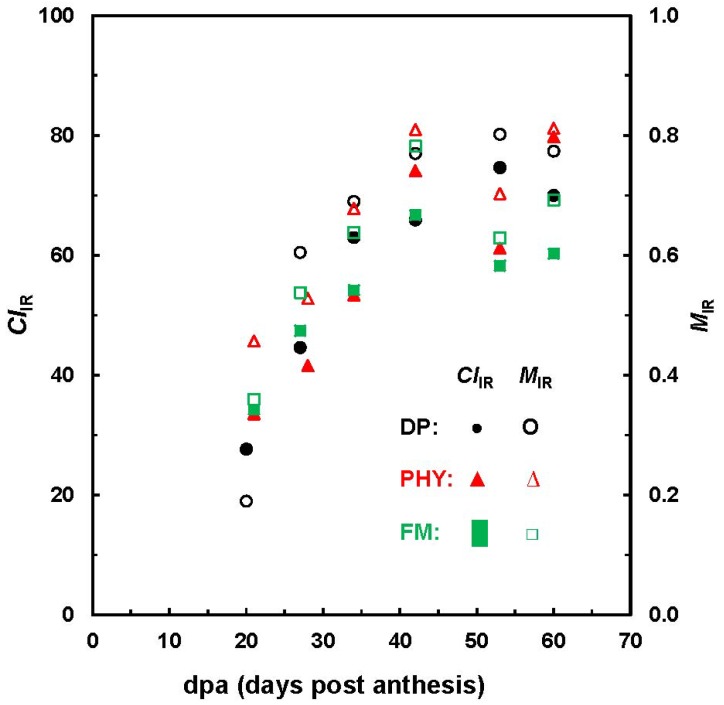
Relationship between *M*_IR_ and *CI*_IR_ values against fiber dpa.

Figure 6 highlights the evolution of *M*_IR_ and *CI*_IR_ with developmental stages. In general, this observation is well consistent with those from XRD analysis on Maxxa and SJ-2 Acala varieties [14]. Hsieh *et al.* reported a steady increase of crystallinity from 38% to 57% between 24 and 60 dpa for Maxxa fibers and a maximum degree of crystallinity (55%) at 34 dpa for Acala fibers. Given the limited number of cotton bolls in this study, it is inappropriate to draw any solid comparisons about the three varieties examined, because fiber growth depends on a number of factors, such as genotype, grown locations and crop managements.

Careful examination of results from different strategies by independent investigators suggests that, in general, immature fibers could have the characteristics of less than 21–28 dpa, *M*_IR_ < 0.58 (in the maturity scale of 0 to 1.0) and *CI*_IR_ < 42% (in the crystallinity scale of 0 to 100%) [11,12,14,16,18]. To interpret the practical samples, a margin of error of 5% to 10% or more should be considered.

### 2.4. Prediction of Cotton Stelometer Fiber Strength

**Figure 7:**
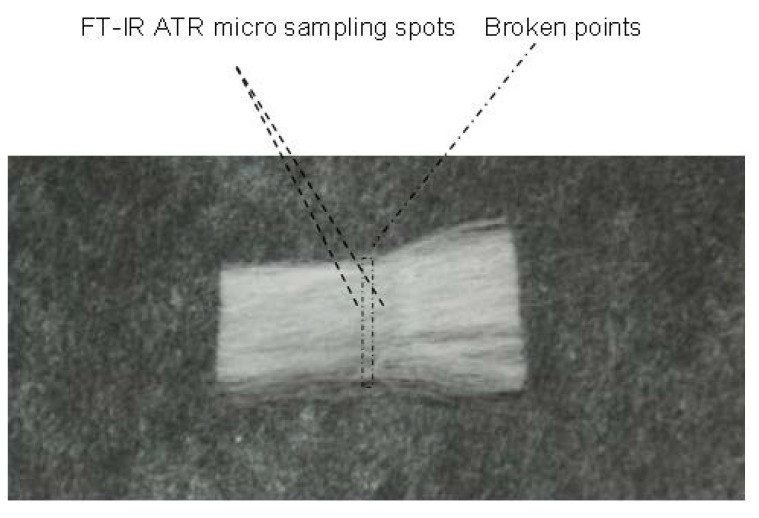
Scheme of broken points to flat bundle fibers from Stelometer strength measurement and ATR-FTIR micro sampling spots (Reprinted with permission from [19]. Copyright 2012 American Society of Agricultural & Biological Engineers).

Strength of cotton fibers is one of several important end-use attributes. To measure cotton strength at the level of either single fiber or flat bundle fibers, a number of testing devices have been in practice. A traditional laboratory testing method known as Stelometer is one of them, which is a different and also complementary tool to the automatic high volume instrument (HVI™) system [19]. With the capability of micro sampling, ATR-FTIR spectra were collected on force-induced broken specimens in fiber bundles (Figure 7) and then were correlated with Stelometer strength (or tenacity) values via partial least squares (PLS) regression [19]. 

By comparing the statistics of optimal results in calibration and validation sets from individual spectral region, Liu *et al.* reported the optimal PLS model performance from the 1800 to 800 cm^−1^ region [19]. However, the resultant spectral model indicated some difficulty in quantitative determination of cotton Stelometer strength for quality control purposes. They attributed this limitation to an apparent lack of uniformity of strength distribution in native fibers and different sampling spot size between spectral (~2 mm in diameter) and Stelometer (~10 mm width × 15 mm length) measurements. To remove the outlier samples that had large differences (or errors) between measured and spectral predicted strength, they applied a 90% confidence interval rule subjectively. As expected, the recalibrated model in the 1800 to 800 cm^−1^ revealed a remarkable improvement in model performance. The result suggested the potential of the ATR-FTIR technique in the quantitative determination of fiber strength. Undoubtedly, more studies are necessary to unravel the strength quality trait and to elucidate the strength mechanism.

Hsieh *et al.* probed the relationship between single fiber strengths and crystalline structures of greenhouse-grown Maxxa and Acala cotton fibers at varying growth stages [14]. They found that single fiber breaking tenacities of Acala fibers do not vary at and beyond 21 dpa, while those of Maxxa cottons appear to be positively related to the secondary cell wall thickening process. They emphasized that, besides the crystallinity and crystallite sizes, other structural parameters, such as fibril orientation and residual stress, may also play key roles in affecting the single fiber strength of cotton fibers. 

### 2.5. Two-Dimensional Correlation Characterization of Fiber Cellulose Development

Two-dimensional correlation (2D-Corr) spectroscopy, a universal and modern technique of vibrational spectral analysis, was originally developed as 2D IR correlation spectroscopy by Noda [20]. Years later, he introduced a more applicable and simple mathematical formalism to perform the generalized 2D-Corr analysis [21], which has been considerably applied to a variety of optical spectroscopic techniques under different types of external perturbations and waveforms. 

Major advantages of 2D-Corr analysis include the enhancement of spectral resolution by spreading peaks over the second dimension, the band assignments through the correlation analysis and probing the complex sequence of events arising from the changes in a system. To obtain 2D-Corr spectra and also to interpret them reasonably, a limited number of spectra were arranged in an increasing or decreasing variable. On the other hand, one of specific challenges might be how to implement the 2D-Corr analysis in large and diverse spectral sets that are common in model development and include multivariate variations in chemical and physical properties. Being a method to explore the variations within a diverse data set, PCA was attempted to classify the spectra of samples [22]. 

**Figure 8:**
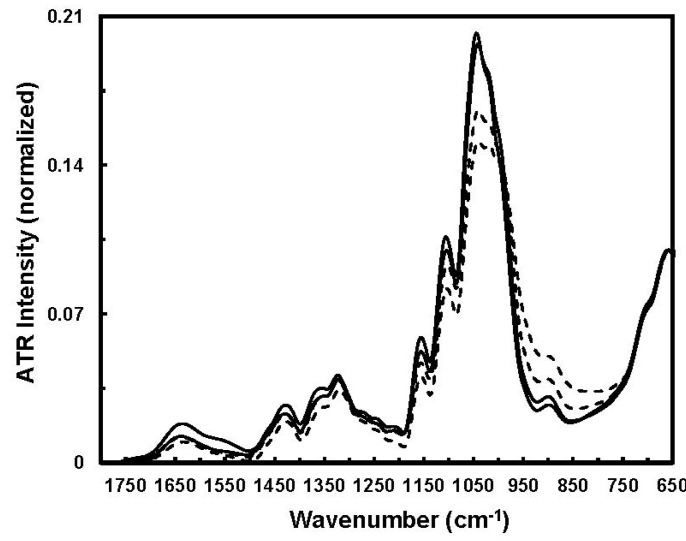
Typical ATR-FTIR spectra of immature (solid line) and mature (dashed line) fibers (Reprinted with permission from [24]. Copyright 2012 Ingentaconnect).

**Figure 9:**
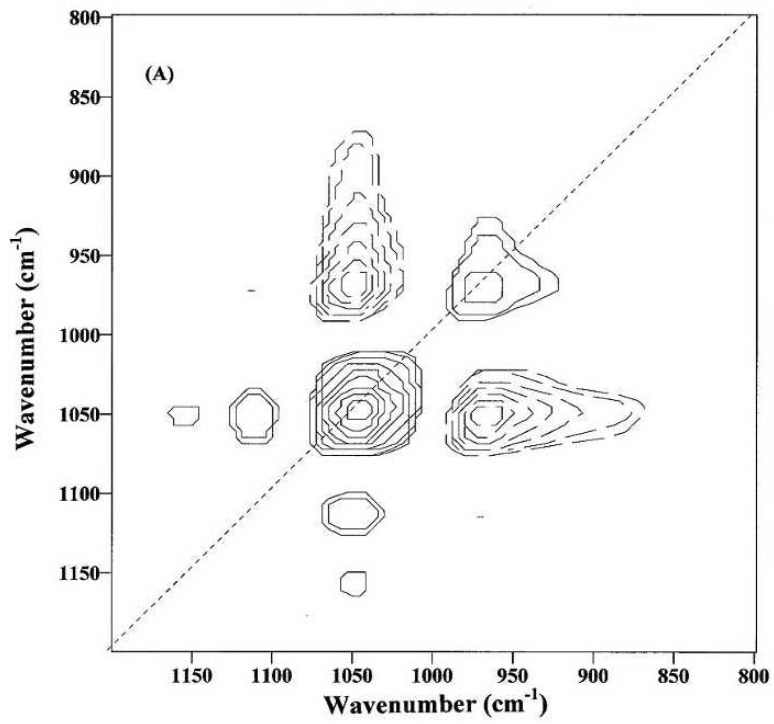
Two-dimensional correlation (2D-Corr) spectrum of immature fibers in the 1200–800 cm^−1^ IR region (Reprinted with permission from [24]. Copyright 2012 Ingentaconnect).

**Figure 10:**
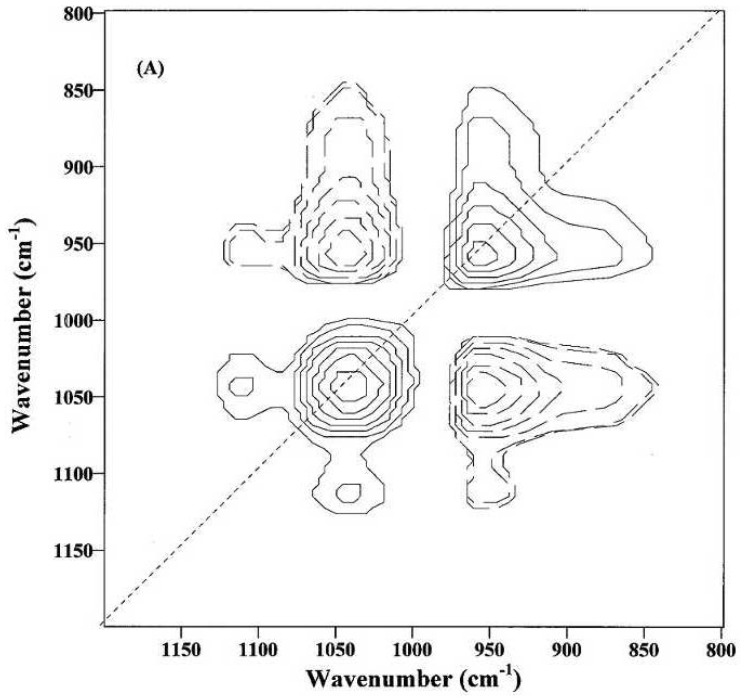
2D-Corr spectrum of mature fibers in the 1200–800 cm^−1^ infrared (IR) region (Reprinted with permission from [24]. Copyright 2012 Ingentaconnect).

Despite the effort of extracting specific information from relatively sharp IR bands of cotton fibers in the 1800–600 cm^−1^ fingerprint region, their spectral features have not been well understood, mainly due to the complexity of IR bands from a mixture of constituents in cotton fibers, slight spectral difference between fiber cellulose and noncellulosic polysaccharides (e.g., sugars), as well as between the amorphous and crystalline celluloses in the 1500–1200 cm^−1^ region [23]. In order to facilitate the interpretation of cotton cellulose IR spectra, Liu *et al.* explored 2D-Corr spectroscopy to analyze the ATR bands in the 1800–650 cm^−1^ IR region for spectral differences and characteristic band assignments between immature and mature fibers [24]. Examples of 2D-Corr spectra of immature and mature fibers are given in Figure 9 and Figure 10, respectively, while their source spectra (or conventional one-dimensional spectra) are shown in Figure 8 for comparison. 

Figure 8 indicates apparent spectral intensity variations in the 1800–650 cm^−1^ region between immature and mature fibers. With respect to cotton growth, cellulose concentrating induces fiber density augmentation and cellulose chain rearrangement through the formation of effective inter- and intra-molecular hydrogen bonding and also inter-chain van der Waals interactions. Clearly, the cellulose chains occur in amorphous and crystalline regions. Hence, a spectral intensity decrease in the 1050–1000 cm^−1^ region might be associated with the functional groups in amorphous regions, and spectral intensity increase in the 1000–950 cm^−1^ region could arise from crystalline regions.

Figure 9 was obtained from immature fibers and might provide information on the compositional and structural changes during the initial fiber development. The appearance of at least two dominant autopeaks (1048 and 968 cm^−1^) at the diagonal position indicates that spectral intensities of these two bands vary greatly with the growth of cotton fibers. The positive cross-peaks (solid lines) at the off-diagonal position are observed between the 1048 cm^−1^ band and the two bands at 1152 and 1110 cm^−1^, while the negative cross-peaks (dotted lines) are found between the 1048 cm^−1^ band and the 968 cm^−1^ band. Hence, the spectral intensity at 968 cm^−1^ increases, while those at 1152, 1110 and 1048 cm^−1^ decrease, as the cotton fibers grow.

With the biosynthesis of cellulose from small and non-cellulosic components (e.g., sugars), fiber cellulose is deposited, and subsequent concentrating might produce a relative increase of crystalline regions and simultaneous decrease of amorphous regions through more effective hydrogen bonding. Apparently, IR absorptions of cellulose chains in amorphous regions differ from those in crystalline regions. To this point, ATR intensity variations observed in Figure 9 are mostly associated with the variations in fiber noncellulosic and cellulosic compositions, which undoubtedly could be presented in more or less the order of amorphous and crystalline regions. Consequently, an increase of spectral intensity at 968 cm^−1^ could be related to the C–O stretching mode of primary alcohols (–C_6_H_2_–O_6_H) in crystalline regions of fiber celluloses. On the other hand, the reduction of spectral intensity at 1048 cm^−1^ might arise from at least one of two sources, either the C–O stretching mode of primary alcohols (–C_6_H_2_–O_6_H) in amorphous regions of fiber celluloses or the C–O vibrational mode of such noncellulosic components as sugars.

An apparent distinction between Figure 10, a 2D-Corr spectrum from mature fibers, and Figure 9, is the appearance of at least two new autopeaks at 1042 and 956 cm^−1^ and also several cross-peaks associated with them. Both autopeaks shift to lower frequencies compared to those at 1048 and 968 cm^−1^ in Figure 9, likely indicating an increase in the cellulose amount that produces more extensive molecular interactions and crystalline portions during this later stage. In the same manner, the reduction of the 1042 cm^−1^ band intensity is positively correlated with the intensity decreases at 1152 and 1110 cm^−1^ and also negatively correlated with the increase in the band intensity at 956 cm^−1^. This suggests that the 956 cm^−1^ band is associated with the C–O stretching mode of primary alcohols (–C_6_H_2_–O_6_H) in crystalline celluloses, and the 1042 cm^−1^ band might come from the C–O stretching mode of primary alcohols (–C_6_H_2_–O_6_H) in amorphous celluloses. 

Two cross-peaks at 1152 and 1110 cm^−1^ in Figure 10 appear at similar positions to those in Figure 9, indicating that these two bands are greatly affected by the amount of cellulose instead of the hydrogen bonding effect that usually causes the shifting in absorption bands. Thus, these two bands could be from amorphous regions, and possibly, the 1152 cm^−1^ band is mainly due to the antisymmetric C–O–C stretching mode of the glycosidic linkage, while the 1110 cm^−1^ band can be attributed to the C–O stretching mode of secondary alcohols (–C_2_–O_2_H). 

In addition, negative cross-peaks between the 1048 cm^−1^ band in Figure 9 or the 1042 cm^−1^ band in Figure 10, with at least one band at 895 cm^−1^ from the 950 to 850 cm^−1^ region, suggest the intensity increase of the 895 cm^−1^ band with fiber development. This is anticipated, since intensity increase of the 895 cm^−1^ band could be indicative of the increase in glycosidic linkage in fiber cellulose. 

## 3. Experimental Section 

### 3.1. Cellulose Formation and Phase Transition

Cotton growth in greenhouse, boll picking, fiber preparation, sugar and cellulose content determination, as well as ATR-FTIR spectral acquisition and interpretation have been described in detail by Abidi *et al.* [11,12].

### 3.2. Discrimination of Immature and Mature Fibers and Assessment of Cellulose Maturity

Two types of cotton samples were used in this study. The first group consisted of a total of 402 mature and immature seed and dried cotton fibers, representing different varieties, locations and crop years. The condition of mature and immature bolls was identified subjectively by their appearance, size and shape. The second group included two sets of lint cottons from diversified sources, and their maturity indices were measured by routine AFIS and IA procedure. All ATR-FTIR spectra of both seed and lint fibers were collected with an FTS 3000MX FT-IR spectrometer, and the data was loaded into Microsoft Excel 2000 to execute simple algorithm analysis [16]. 

### 3.3. Determination of Cellulose Crystallinity

Eighteen seed and dried cotton bolls from 3 cultivars (Delta pine—DP, Fibermax—FM and Phytogen—PHY) were collected at 6 developmental stages of 21 through 60 dpa. The ATR-FTIR spectra of all 18 bolls were acquired with an FTS 3000MX FT-IR spectrometer, and XRD data of only 10 bolls were obtained using a Rigaku Ultima IV Diffractometer. Two data sets were exported into Microsoft Excel 2000 for simple algorithm analysis [18]. 

### 3.4. Prediction of Cotton Stelometer Fiber Strength

Stelometer bundle strengths (or tenacities) of lint cottons in great diversities were obtained by following the standard protocol. The ATR-FTIR spectra from broken specimens were collected and then spectral response was correlated with Stelometer strengths through PLS regression in the PLSplus/IQ package [19]. 

### 3.5. Two-Dimensional Correlation Characterization of Fiber Cellulose Development

The ATR-FTIR spectra of immature and mature seed cottons were simply offset to zero at the wavenumber of 1800 cm^−1^ and then were loaded into the PLSplus/IQ package for preliminary PCA characterization. Next, normalized spectral sets representing immature and mature fiber clusters were created with the ordered sequence of increasing PC1 scores, and subsequent 2D-Corr analysis was conducted [24].

## 4. Conclusions

Cotton fiber cellulose is accumulated during the secondary cell wall biogenesis. Both cellulose content measurement and the ATR-FTIR spectroscopic technique are suitable to monitor the different transition phase between the primary wall and the secondary cell wall for two cultivars, with TX19 fibers occurring between 17 and 18 dpa and TX55 fibers between 21 and 24 dpa. 

Comparison of comprehensive observations on developmental fibers from cellulose content measurement, PCA pattern, *M*_IR_ and *CI*_IR_, as well as *CI*_XRD_ determination, indicates that, in general, immature fibers could be distinguished by the characteristics of less than 21–28 dpa, *M*_IR_ < 0.58 (in the maturity scale of 0 to 1.0) and *CI*_IR_ < 42% (in the crystallinity scale of 0 to 100%). 

The 2D-Corr characterization clearly indicates the IR intensity increase or decrease of the bands ascribed to different C-O confirmations of primary alcohols from immature to mature fibers in the 950–1050 cm^−1^ region. Intensity increase of the 956 cm^−1^ band in the 900 and 1000 cm cm^−1^ region, together with the intensity decreases of several bands in the 1000 and 1100 cm^−1^ region, is a mainstream of cotton fiber development and maturation. These unique bands could be useful in interpreting two key bands at 1032 and 956 cm cm^−1^ that were utilized to develop a simple algorithm for the classification of immature fibers from mature ones.

Apparently, the algorithm approach in ATR-FTIR spectral sensing is the most attractive and interesting, since, in its simplest form, there is no calibration model that is commonly built from a large sample set. In addition, the use of intensities at unique wavenumbers could reduce the influences from other components in diverse samples; hence, it could be universally applied for fast, accurate, routine and direct determination of cotton fiber maturity and crystallinity, simultaneously. This procedure avoids the need to perform any pretreatments of cotton fibers, analyzes a small amount of fibers (as little as 0.5 mg) and requires only a short time (less than 2 min) for sample loading, spectral acquisition and subsequent data reporting. 
